# Correlative X-ray imaging to reveal the dissolution of nanoparticles and nutrient transport in plant foliar fertilization

**DOI:** 10.3389/fpls.2025.1610402

**Published:** 2025-06-24

**Authors:** Chengpeng Wu, Emil V. Kristensen, Francesco Minutello, Augusta Szameitat, Søren Husted, Rajmund Mokso

**Affiliations:** 1 Department of Physics, Technical University of Denmark, Lyngby, Denmark; 2 Plant and Environmental Sciences, University of Copenhagen, Frederiksberg, Denmark

**Keywords:** SAXS, XRF, Micro-CT, plant imaging, foliar fertilization, dissolution of nanoparticles, nutrient transport

## Abstract

The integration of nanotechnology in agriculture allows for more precise nutrient delivery through nanoparticles (NPs), particularly via foliar application. To mature this technology for enhancing fertilizer efficiency, it is essential to shed new light on the transport and dissolution of NPs in plants. Available analytical methods struggle to address this challenge in a direct manner. We introduce correlative X-ray imaging as a novel analytical tool capable of tracking NP pathways, dissolution and hence nutrient release in plants. By utilizing three complementary X-ray techniques, we offer a unique insight into the plant processes associated with foliar fertilization. We demonstrate that small-angle X-ray scattering enables the characterization of NP size and concentration, while X-ray fluorescence imaging, maps the distribution of elements within the sample. Finally, micro-computed tomography integrates these findings into a complete three-dimensional digital representation of the plant’s microstructure, revealing regions of apparent densification associated with NP accumulation. Using freeze-dried barley plants infiltrated with nano-hydroxyapatite (nHAP), we observed rapid dissolution of NPs, and we are able to associate time and space attributes to the translocation process of nutrients up to three days following foliar application of NPs. With the first pilot study of applying correlative X-ray imaging to live plants, we sought to indicate the potential of this new analytical approach for future nano-enabled agricultural research.

## 1 Introduction

Ensuring stable agricultural food production for the global population remains a significant challenge. Although current agricultural systems may be capable of supplying adequate caloric intake for today’s population, sustaining this level of production for a rapidly growing population in the future presents a critical concern. While fertilizer application is essential for food production, current practices remain inefficient and environmentally unsustainable, especially due to their impact on climate change and pollution. The use of nitrogen (N) and phosphorus (P) substantially exceeds planetary boundaries (Kopittke et al., 2021), underscoring the urgent need to improve nutrient use efficiency to enable the sustainable intensification of agricultural production (Wang et al., 2021; Langhans et al., 2022).

A promising approach might be to use foliar fertilization, in which nutrients are applied directly to plant foliage. This method can bypass microbial and chemical fixation in the soil-plant system, potentially enhancing nutrient uptake efficiency (de Castro and Schjoerring, 2024). However, the direct foliar application of soluble nutrients is constrained by several factors, including the risk of leaf scorching due to high ionic concentrations and low solution pH, as well as the low translocation capacity of many foliar-applied nutrients (McBeath et al., 2020). These limitations necessitate frequent low-dose applications, which may render the practice economically unfeasible. As an alternative, foliar application strategy, tailoring the essential plant nutrients into NPs for efficient plant uptake, has the potential to increase the efficiency of fertilization (Husted et al., 2023; Lowry et al., 2019). Due to their small size (*<*100 nm), NPs have the possibility of overcoming several plant physiological barriers, including cuticular penetration, phloem loading, and translocation to developing tissues. Moreover, the design of NPs for slow dissolution enables controlled and continuous nutrient release, reducing the frequency of application required over the growing season.

Numerous studies have highlighted the potential of nanomaterial-based fertilizers to enhance plant nutritional status (Kah et al., 2019; Kang et al., 2021; Gao et al., 2021; Lowry et al., 2024). For example, Kah et al. (2019) discussed how nanoparticulate delivery platforms could improve nutrient use efficiency and targeted agrochemical application, while also noting critical challenges in understanding NP transformation and transport within plants. Kang et al. (2021) demonstrated that silica NPs with tunable dissolution rates could improve biomass and disease resistance by facilitating silicon uptake. Gao et al. (2021) further showed that encapsulating ZnO NPs within mesoporous SiO_2_ shells enabled sustained micronutrient delivery via foliar application. Despite these advances, the underlying mechanisms governing NP uptake, translocation, dissolution, and the eventual assimilation of their ionic constituents into plant tissues remain insufficiently understood. As emphasized by Lowry et al. (2024), developing new tools to rapidly and accurately characterize nanoparticle–plant interactions is critical for advancing nano-agriculture and enabling precise, efficient, and sustainable nanocarrier-based delivery strategies. While a range of analytical techniques has been employed to detect and trace NPs in plant systems (Lv et al., 2019; Azim et al., 2023; Zhang and Su, 2024), each method presents inherent limitations. High-resolution imaging tools such as scanning electron microscopy (SEM) and transmission electron microscopy (TEM) allow for detailed visualization of NPs, but are restricted to small sample areas and rely on sample preparation procedures that may inadvertently displace or dissolve NPs. Elemental analysis techniques, including inductively coupled plasma mass spectrometry (ICP-MS) (Avellan et al., 2019) and energy-dispersive X-ray spectroscopy (EDS) (Hodoroaba, 2020; Li et al., 2021), offer high sensitivity for elemental detection. However, they typically provide only localized elemental data rather than large-area distribution patterns. Furthermore, nano-scale artifacts may be misidentified as NPs unless EDS probes are used to confirm their elemental composition. These challenges are particularly pronounced in hydrated soft tissues (Tiede et al., 2008). Three-dimensional imaging techniques such as X-ray computed tomography (Piovesan et al., 2021; Kristensen et al., 2025), and confocal laser scanning microscopy (CLSM) (Avellan et al., 2019; Santana et al., 2020) facilitate non-destructive, *in vivo* imaging of NP distributions. However, these techniques often lack the sensitivity needed to detect NPs at low concentrations, thus requiring the use of NP concentrations higher than those typically encountered under field conditions. To address these limitations, we propose a correlative X-ray imaging approach that integrates the high-resolution capabilities of microscale imaging with the broader context provided by wide-field visualization. This method enhances sensitivity, enabling the detection of NPs at low concentrations and supporting a more accurate and comprehensive understanding of NP behavior and elemental distribution within plant tissues over time.

**Figure 1:**
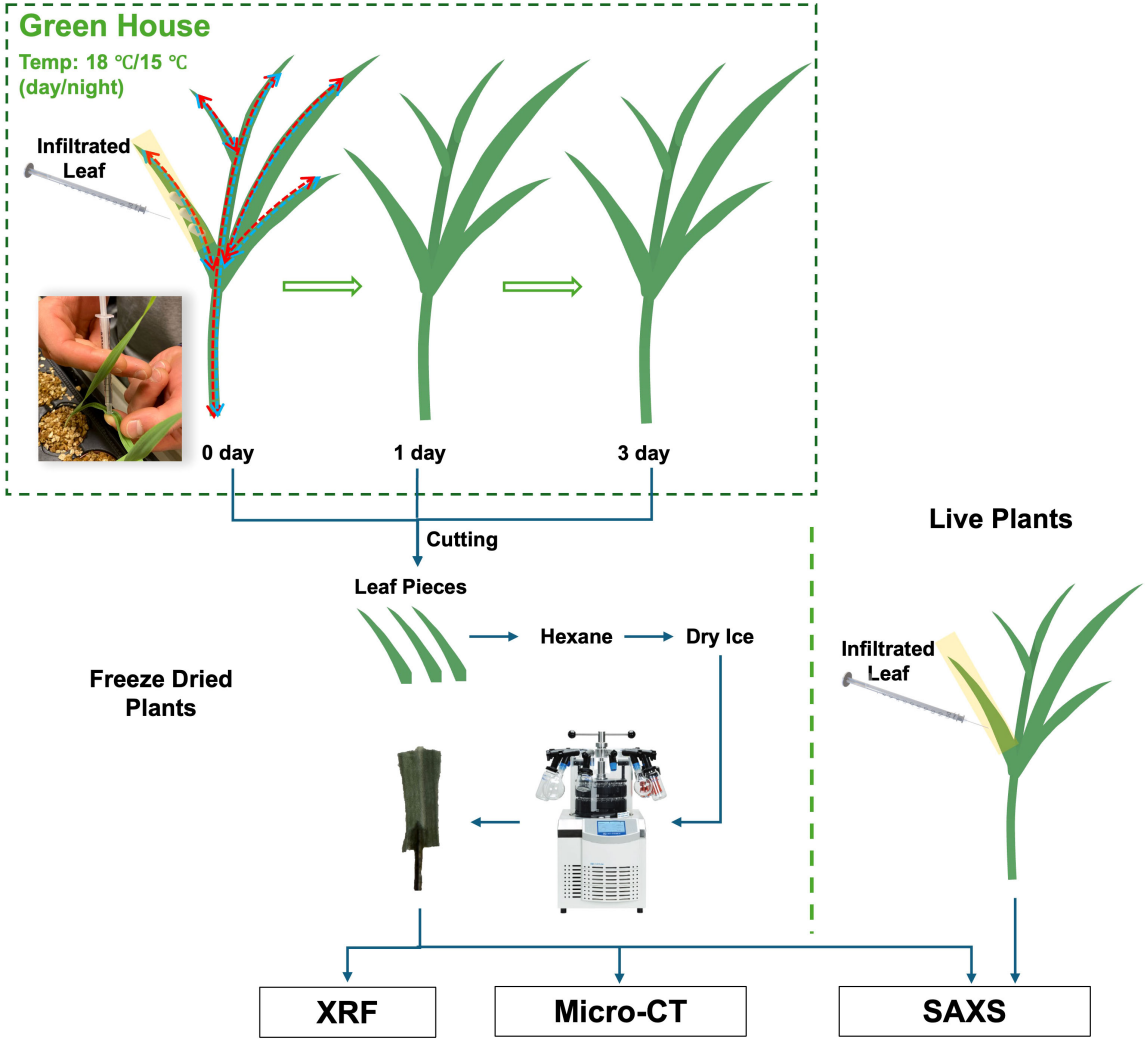
Schematic diagram of the entire process from plant sample cultivation to X-ray imaging.

To the best of our knowledge, this study represents the first application of small-angle X-ray scattering (SAXS) imaging to directly visualize NPs embedded within plant tissues and to monitor changes in their structure and distribution. SAXS is an established X-ray technique for probing materials structure at the nanometer scale. The signal is generated by scattering of X-rays due to electron density differences between particles and their surrounding medium (Narayanan, 2024). SAXS signal variations can provide insights into the *in planta* fate of NPs, including processes such as aggregation, dispersion, dissolution, or translocation. However, distinct physicochemical processes can result in similar scattering signatures, giving rise to possible false interpretations. To overcome this limitation of a single method, we integrated three complementary X-ray methods: SAXS, X-ray fluorescence (XRF) and micro-computed tomography (Micro-CT) as depicted in **Figure 1**. With XRF we map the element distribution, with Micro-CT we visualize the 3D microstructure and combined with SAXS we build a comprehensive understanding of NP behavior within plant tissues.

Since no prior studies have used SAXS to image the NPs examined in this work, we first conducted SAXS imaging on NP-only solutions to establish a baseline for sensitivity. Once the distinct NPs scattering signals were established, we applied a freeze-drying protocol in which leaves infiltrated with the nHAP solutions with the strongest scattering signal were frozen at specific intervals, to capture the NP distribution and dissolution state at each time point. Freeze-dried leaf samples were then subjected to synchrotron SAXS imaging, followed by Micro-CT imaging and elemental XRF mapping. To prove its long term potential, we conducted the first scanning SAXS imaging experiments on live plants with encouraging outcome.

## 2 Materials and methods

In this section, we describe the materials and methods used in the approach proposed in this study. In Section 2.1, we introduce the types of NPs involved, as well as the procedures for plant sample cultivation and pre-imaging preparation. In Sections 2.2 to 2.4, we detail the experimental setups and imaging conditions corresponding to the three X-ray imaging methods used in this study.

### 2.1 Nanoparticles and plant samples

This study primarily involves three types of NPs, including hydroxyapatite (nHAP), mesoporous silica with ZnO (ZnO@MSN), and manganese oxide (MnO), selected for their relevance in the field of nanofertilization (Hadri et al., 2024). The chemical compositions and structural characteristics of the NPs used in this study are summarized in **Table 1**. Particle synthesis and characterization are described in Burve et al. (2024); Gao et al. (2021); Pinna (2024); Szameitat et al. (2021). To enhance the X-ray imaging contrast of nHAP particles, we employed a spiking strategy by incorporating two heavy metal elements, cerium (Ce) and europium (Eu), into their structure.

**Table 1:** Chemical compositions and structural parameters of the NPs used in this study.

Name	Components	Geometric parameters	Description
nHAP-1	Ca10(PO4)6(OH)2	Length: 28 nm; Width: 16 nm	Crystalline nano-hydroxyapatite
nHAP-2	Ca10(PO4)6(OH)2+ Ce + Eu	Length: 40 ± 12 nm; Width: 13 ± 5 nm	Crystalline nano-hydroxyapatite doped with cerium and europium
ZnO@MSN	SiO2 + ZnO	Core radius: 33 nm; Shell thickness: 12 nm	ZnO encapsulated in mesoporous SiO2
MnO	(C3H4O2)n + MnO	Radius: 12 nm	poly(acrylic acid)-coated Mn oxide

In the table, nHAP-1 is used in the solution experiments in Section 3.1, while nHAP-2 is used in the freeze-dried leaf experiments in Section 3.2.

To gain insights into the contrast and expected SAXS curves of the NPs within plant tissues, we included the scattering curve of a solution containing Nicotiana tabacum BY-2 suspension cells, a well-established plant cell model. This comparison helps in understanding how the scattering signals from NPs might differ when measured within the complex matrix of plant tissues. The BY-2 cells were washed with BY-2 medium (pH 6.35), and the final pH of the suspension was adjusted to 5.92. A 1:1 dilution was performed by adding 4 mL of BY-2 medium to the settled cells.

Barley seeds (*Hordeum vulgare L.* cv. KWS Irina) were germinated in vermiculite for 7 days. After germination, seedlings were transplanted to aerated 5L containers with hydroponic solution. The hydroponic solution was changed weekly and contained KH_2_PO_4_ (200 *µ*M), K_2_SO_4_ (200 *µ*M), MgSO_4_·7H_2_O (300 *µ*M), NaCl (100 *µ*M), Mg(NO3)_2_·6H_2_O (300 *µ*M), Ca(NO_3_)_2_·4H_2_O (900 *µ*M), KNO_3_ (600 *µ*M), Fe(III)–EDTA–Na (50 *µ*M), H_3_BO_3_ (2 *µ*M), Na_2_MoO_4_·2H_2_O (0.8 *µ*M), ZnCl_2_ (0.7 *µ*M), MnCl_2_·4H_2_O (1 *µ*M) and CuSO_4_·5H_2_O (0.8 *µ*M). The pH of the nutrient solution was maintained at 5.0-6.0 using HCl. Plants were grown in a greenhouse environment with minimum day/night temperatures of 18 C/15 C and a 16-hour day, 8-hour night cycle with minimum light intensity (PAR) 300 *µ*mol*/*(m^2^·s). 14 days after transplanting, the youngest fully evolved leaves were infiltrated with NP solutions using a disposable plastic syringe without a needle. Following infiltration, the plants were grown under the same conditions and harvested at 0, 1, and 3 days after treatment. Immediately after harvest, 5 mm × 10 mm segments were excised from the infiltrated leaves, submerged in hexane at -80 °C (cooled with dry ice), and then lyophilized using a freeze-dryer (Christ Alpha 1-4, Germany) at 1 mbar and -40 C. Samples were stored in sealed containers until imaging.

### 2.2 SAXS experiments

X-ray scattering is typically applied when nanometer sized constituents of a sample are to be characterized in mm to cm sized samples. The signal is produced by the interaction of the electromagnetic X-ray wave with the electron clouds in the sample. This is complementary to the attenuation induced effects typically forming the image in conventional X-ray tomography. The magnitude of scattering contrast is proportional to the atomic number and density of the material, while variations in particle size are reflected in the angular intensity profile: smaller particles contribute to scattering at larger angles.

The SAXS experiments were performed at the ForMAX beamline of the MAX IV Synchrotron in Lund, Sweden, equipped with an Eiger2 4M detector (Nygård et al., 2024). Freeze-dried samples were mounted in the sample holder using super glue and a pin making sure the region of interest on the sample was just above the pin for maximum stability of the sample. Live plants were placed in an environmental chamber with leafs positioned with the flat side facing the X-ray beam direction. The measurements were conducted at standard room temperature and humidity. The selected beam energy was 20.129 keV, with a focused beam spotsize of 50 × 50 *µm*^2^. The setup used allows scanning the *q*-range from 0.00015 to 0.18 Å^−1^. The exposure time was 100 ms for both the solution and freeze-dried sample experiments, while it was 50 ms for the live plant experiments.

The typical scanning area for each axis of a sample was ranging from 5 to 15 mm. The smallest sample required scanning 100 × 100 sampling points, while the largest samples required 300 × 150 points. Therefore, the total acquisition time for one complete scan ranges between 8 and 40 min.

**Figure 2:**
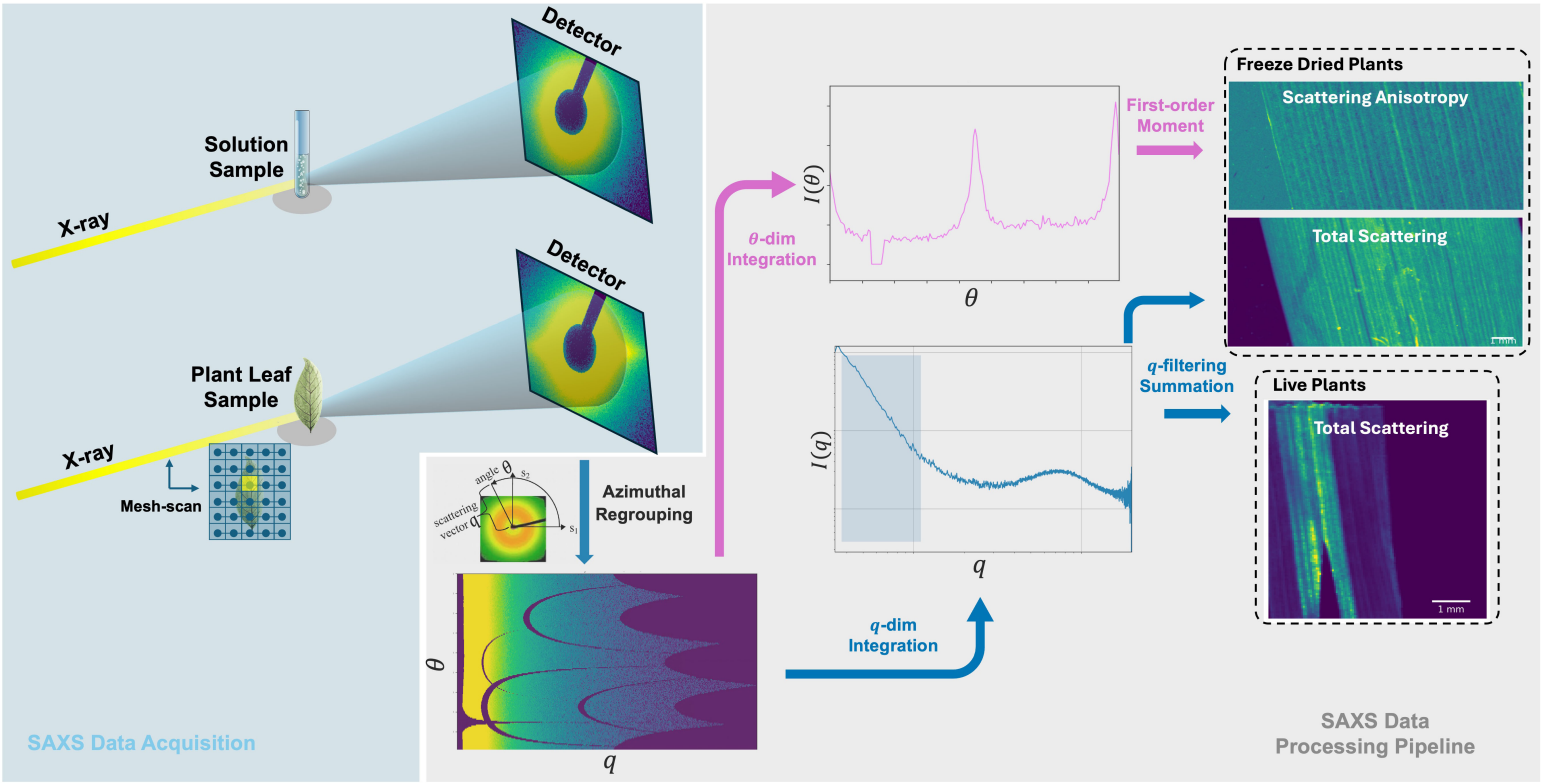
Schematic diagram of SAXS experimental data acquisition and processing pipeline. In the data acquisition part, isotropic scattering patterns are typically observed for solution samples, while anisotropic scattering patterns appear for plant samples due to the internal cellulose structure. In the data processing part, the raw detector data is first azimuthal regrouped, followed by integration along two dimensions: the scattering vector *q* and the azimuthal angle *θ*. Finally, the total scattering intensity and scattering anisotropy are obtained through filtering and summation as well as first-moment calculation, respectively. The computed results from all mesh-scan points are then assembled to generate the final two feature maps. For live plants, the scattering signal of cellulose is very close to that of some NPs, while the scattering anisotropy can distinguish them. For freeze-dried samples, the scattering signal of NPs is much stronger and therefore, total scattering map is enough to show the results.

At the ForMAX beamline, all raw SAXS measurement data were processed using the MatFRAIA method implemented in Python for radial and azimuthal integration (Jensen et al., 2022). The data were compressed into one-dimensional scattering curves *I*(*q*) and two-dimensional forms *I*(*q,θ*), where *q* represents the scattering vector and *θ* represents the azimuthal angle. For solution samples, we also scanned empty tubes and tubes filled with water for scattering signal correction. By selecting a range along the sample with relatively uniform longitudinal distribution and a scattering scale close to the size of the NPs of interest, the final effective q-range was determined to be 0.004 to 0.022 Å^−1^. As shown in **Figure 2**, for the two-dimensional scattering data *I*(*q,θ*), in addition to performing one-dimensional integration along the scattering vector dimension, we also evaluated the total scattering map by summing the raw scattering information at each scanning point of the mesh-scan. For freeze-dried plants, the total scattering map is enough to analyze the signal trend, while for live plants, we also performed one-dimensional integration along the azimuthal dimension to obtain anisotropic scattering curves. The first moment was calculated as a characterization of scattering anisotropy, $M_{1}(\theta )\;=\int \theta \cdot I(\theta )d\theta$, and then we also obtained the scattering anisotropy map by summing the first moments for mesh-scan.

### 2.3 Micro-CT experiments

The Micro-CT experiments were conducted by using the Exciscope Polaris microtomographic scanner located at the 3D imaging center of the Technical University of Denmark. The X-ray source in the Polaris system is a liquid metal jet from Excillum. The detector is a CMOS camera with 4k × 4k pixels of 16 *µ*m size. During the scanning process, the X-ray source operated at a voltage of 40 kV and a power of 85 W. The distance from the source to the detector was set to 92 cm, while the distance from the source to the object’s rotation center was 16 cm, resulting in a magnification factor of 5.8 and an effective voxel size after X-ray magnification of 2.78 *µ*m. This configuration also results in an effective propagation distance of 13 cm. This allows the formation of a near field diffraction pattern on the detector, enhancing the attenuation contrast by the so called phase contrast component. This component can be utilized to significantly boost the image contrast between materials of similar density. The first processing step therefore consisted in phase retrieval using the homogenaous material approximation by Paganin (Paganin et al., 2002). The resulting, filtered angular projections are then assembled into a 3D tomogram using cone-beam tomographic reconstruction algorithm. The 3D images then undergo quantification using our own algorithms in python and visualized using Dragonfly software (Comet Technologies Canada Inc., Montreal, Canada, 2024).

### 2.4 XRF experiments

Micro-resolution XRF mapping experiments were performed using the Bruker Tornado M4+ micro-XRF scanner at the X-ray laboratory of the Department of Geosciences and Natural Resource Management, University of Copenhagen. The X-ray source was operated at 50 kV acceleration voltage and 600 *µ*A current. The X-ray beam was focused to a 20 *µ*m spot size on the sample using focusing optics. Due to uneven sample surfaces, the depth focus was adjusted to the midpoint between the top and bottom of each sample. Spectroscopy maps of freeze-dried leaves were collected by scanning with a 20 *µ*m pixel size and 20 ms dwell time per pixel. Spectroscopy maps were fitted to selected elements using Bruker’s Tornado software, successfully fitting the most important elements including K, P, Ca (K-edges), and Ce and Eu (L-edges). These maps provided sufficient signals for the most relevant materials (P and Ce) of the dosed NPs. Given the relatively high energy of the incident X-rays and the low sample density, this method probed the entire sample thickness simultaneously.

## 3 Results

### 3.1 Linking SAXS signal to NP concentration

**Figure 3:**
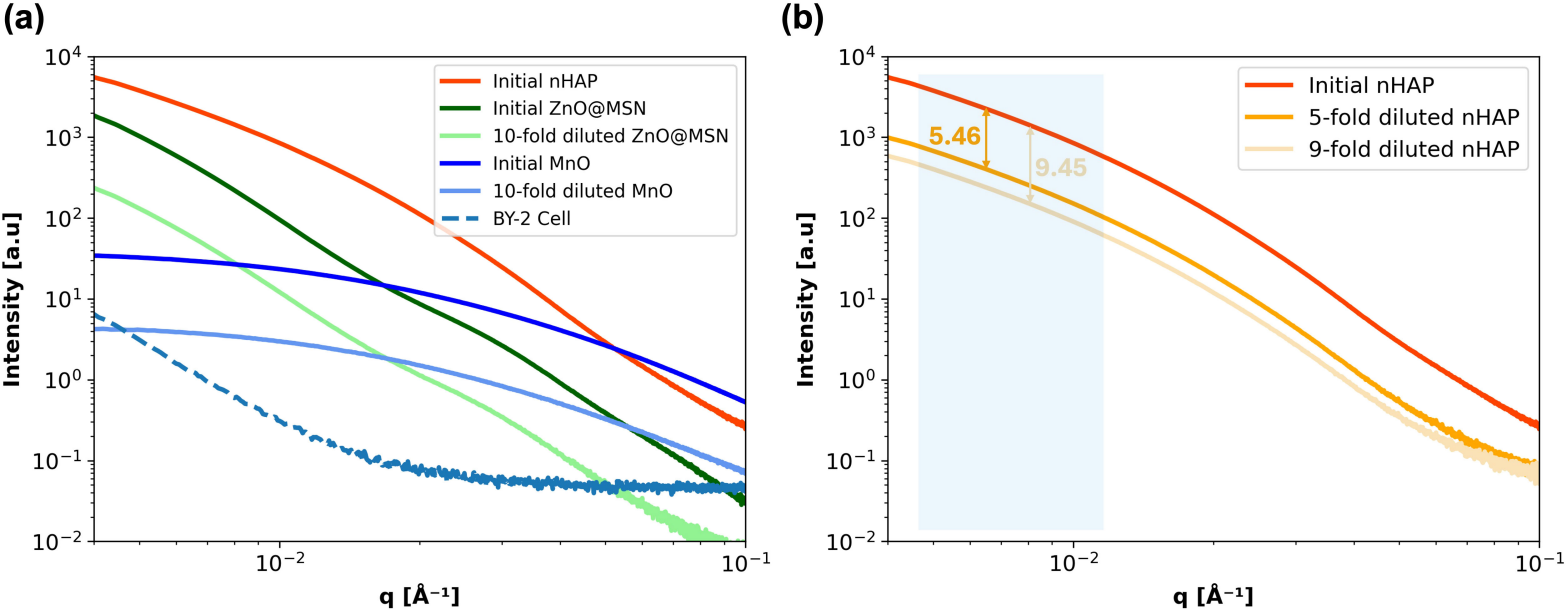
**(a)** The SAXS curves at initial concentration and 10-fold diluted ZnO@MSN and MnO solutions with the reference BY-2 cell solution. **(b)** The SAXS curves of different diluted rates of initial concentrations (**Table 1**), 5-fold and 9-fold diluted nHAP solutions.

Plant tissue has various structural components that are good scatterers when it comes to the interaction with an X-ray beam. The first general task is to separate the SAXS signal originating from the native plant structure and the regions containing the NPs. To do this, we must establish the relationship between the scattering intensity from several types of NPs and the sensitivity of SAXS signals at different concentrations in comparison to the scattering curve of plant cell solutions. **Figure 3** illustrates the SAXS characteristic curves for three model NPs (*i.e.*, nHAP, ZnO@MSN, and MnO) and for the reference BY-2 cells measured in solution. For nHAP, results are shown for the initial concentration 2.5 g/L (**Table 2**), then 5-fold diluted, and 9-fold diluted solutions. For ZnO@MSN and MnO, two configurations were tested: the initial concentration 4 g/L and 2.6 g/L (**Table 2**) and 10-fold diluted.

**Table 2:** The fitting results of different diluted rates of nHAP, ZnO@MSN, and MnO solutions.

NP	Concentration	*R_g_*[Å]	*I*0	Reduction ratio of *I*_0_
nHAP	∼ 2.5 g/L	297 ± 2.86	7845 ± 148	Baseline
	5-fold	298 ± 2.84	1437 ± 27.9	5.46
	9-fold	296 ± 2.90	830 ± 17.4	9.45
ZnO@MSN	∼ 4 g/L	367 ± 6.30	3120 ± 181	Baseline
	10-fold	365 ± 19.2	393 ± 70.6	7.93
MnO	∼ 2.6 g/L	109 ± 0.79	35.0 ± 0.141	Baseline
	10-fold	109 ± 1.50	4.46 ± 0.0412	7.84

**Figure 3** shows that nHAP at initial concentration produces the strongest scattering signal, followed by ZnO@MSN and MnO, all exceeding the reference BY-2 cell signal. With the relatively low total scattering values of the MnO particles these are judged to be less optimal for *in planta* experiments than the nHAP and ZnO@MSN particles both providing ample contrast, especially at low *q*-ranges. Additionally, the scattering characteristic curves of the three NPs maintain consistent shapes after dilution, with only a reduction in signal intensity indicating a minimum of interparticle interaction.

For quantitative analysis of the raw NP solutions, we performed a simple Guinier’s approximation fitting using the BioXTAS RAW2 software (Hopkins, 2024). **Table 2** lists the two primary parameters obtained from the fitting, *R_g _*and *I*_0_. *R_g_* represents the gyration radius, which reflects the average particle size in the solution, while *I*_0_ is the intensity at zero scattering angle, indicating the particle concentration in the solution. As shown in **Table 2**, *R_g_*remains nearly constant across solutions with different concentrations of the same type of NPs, indicating that the particle size is stable before dissolution happens. In contrast, *I*_0_ decreases as the particle concentration drops, which aligns with expectations. In the last column of **Table 2**, we calculated the reduction ratio of *I*_0_ after dilution compared to its initial concentration. This ratio shows a strong positive correlation with the dilution factor. For nHAP solutions, the error between the *I*_0_ ratio and the dilution rate is less than 10%, indicating that SAXS signals are highly sensitive to low concentrations of NPs and can effectively distinguish them from background scattering signals.

### 3.2 nHAP dissolution in freeze-dried leaf

**Figure 4:**
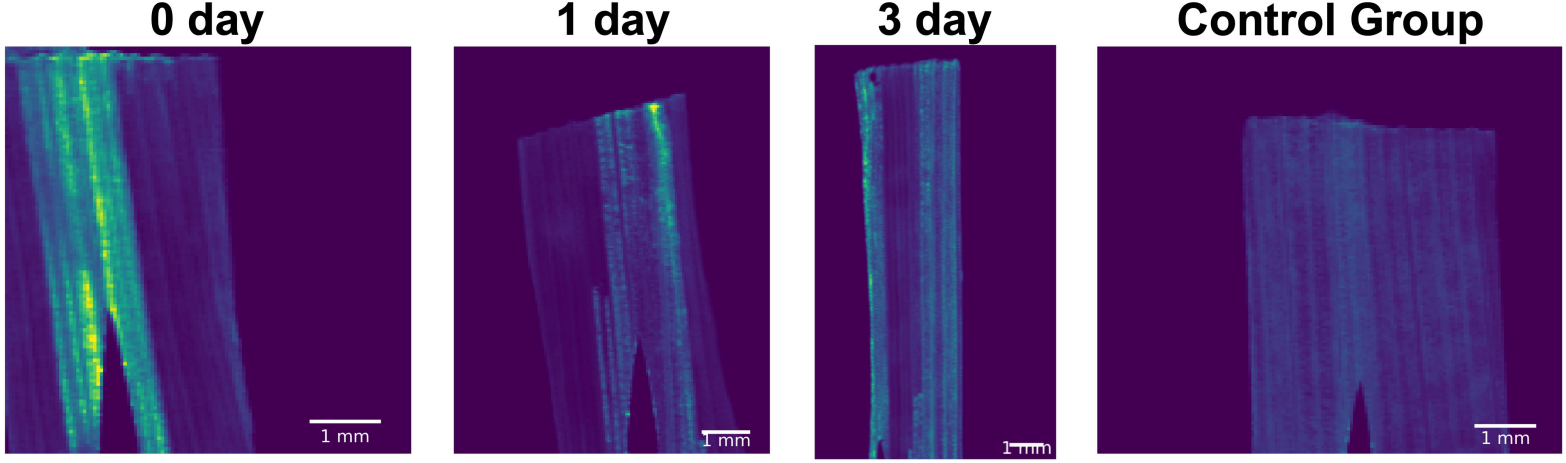
The total scattering intensity maps of the freeze-dried leaves infiltrated with nHAP solutions after different treatment times, and one of the control group just infiltrated with citrate solution. All images are displayed with identical range of the color map.

**Figure 4** presents the total scattering intensity of samples freeze-dried on the day of nHAP infiltration (day 0), and after one day (day 1) and after three days (day 3) post-infiltration, along with results from the control group. Compared to the three groups of samples infiltrated with nHAP, the control group exhibited significantly lower and more uniform SAXS signal intensity, primarily originating from the nearly parallel cellulose structure, with no distinct additional feature regions. A marked decrease in scattering intensity was observed by day 1, followed by a less pronounced decrease between day 1 and day 3. SAXS signals primarily reflected the size and concentration of NPs. When the particle size remained relatively stable, a decreased signal indicates a reduction in particle concentration, suggesting that NPs have either dissolved or translocated. To further distinguish between dissolution and translocation, we can examine the distribution of main elements in NPs using XRF. If the elemental content remains largely unchanged, it provides additional evidence that NPs have dissolved with the elements merely transformed into their ionic forms, but not translocated.

**Figure 5:**
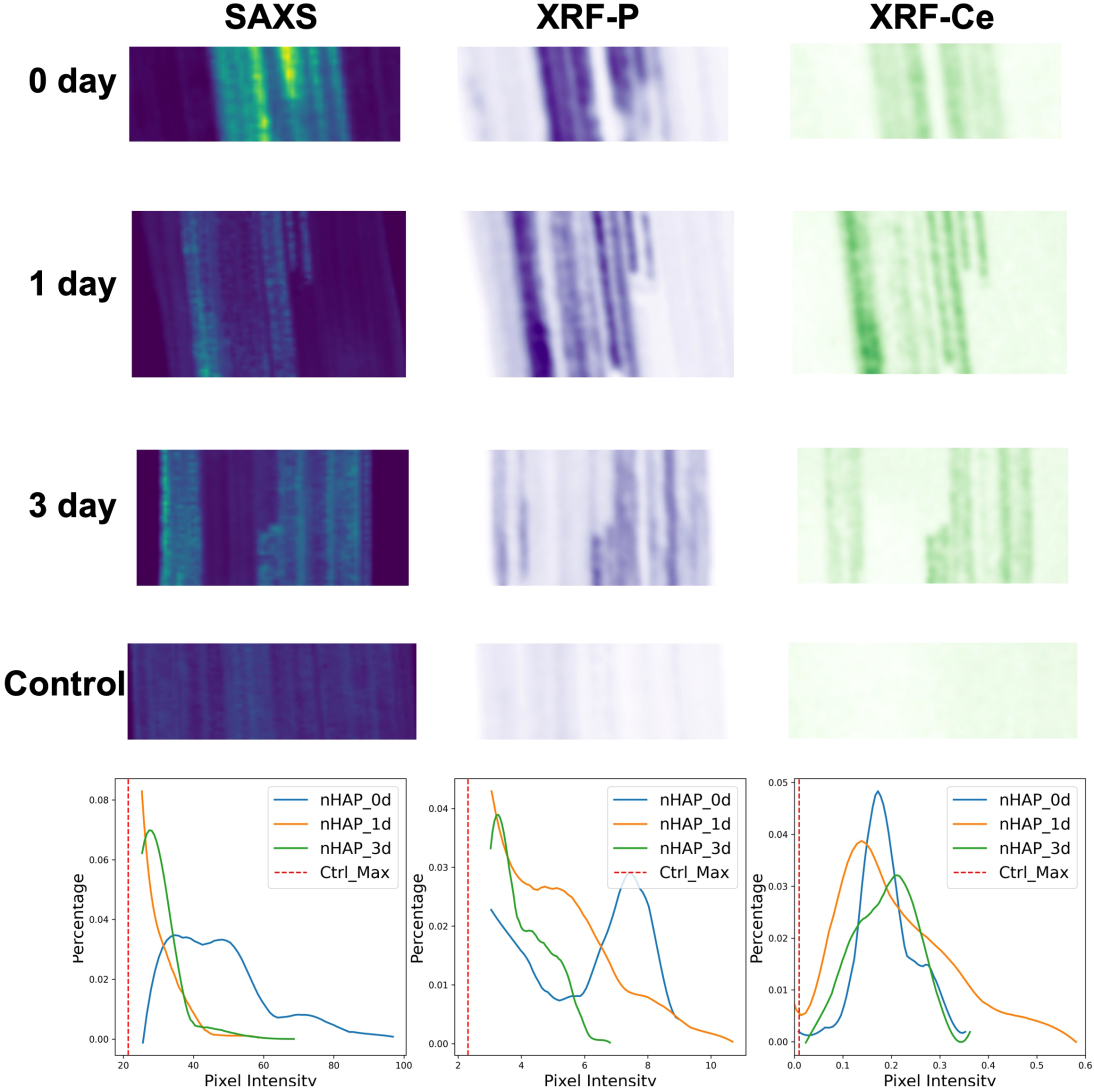
The aligned total scattering intensity maps and XRF intensity maps for P and Ce elements of the freeze-dried leaves infiltrated with nHAP solutions after different treatment time, and one of the control group just infiltrated with the citrate solution. The bottom row shows the corresponding statistical distribution histograms for SAXS, XRF-P, and XRF-Ce signals. Since the control group signals without NPs are primarily concentrated on the left side, only the maximum signal value of the control group is plotted as a reference to focus on the NP distribution region.

In **Figure 5**, the SAXS images are correlated to the XRF mapping data of P and Ce of the same samples. For technical reasons, the alignment with the original SAXS images could not be perfectly maintained, and therefore we cropped a partial region of the sample to align these images. In this way, we were able to visualize consistent trends across the three time points and the three corresponding maps. In the SAXS modality, the day 0 sample yields a clear peak at high pixel intensities as seen in the histogram of **Figure 5**, both the day 1 and day 3 samples show more counts at lower pixel intensity indicating that the highly scattering particles present in the day 0 sample have been partially dissolved. For the P signal of XRF, a similar behavior is seen with a clear defined peak at high pixel values at the day 0 sample and less high pixel values for day 1 and day 3. Contrary for the Ce XRF maps, the histograms exhibit a peak not moving significantly in value between the different time points. From these three modes it can be seen that the particles themselves must dissolve as the SAXS signal decays over time.

**Figure 6:**
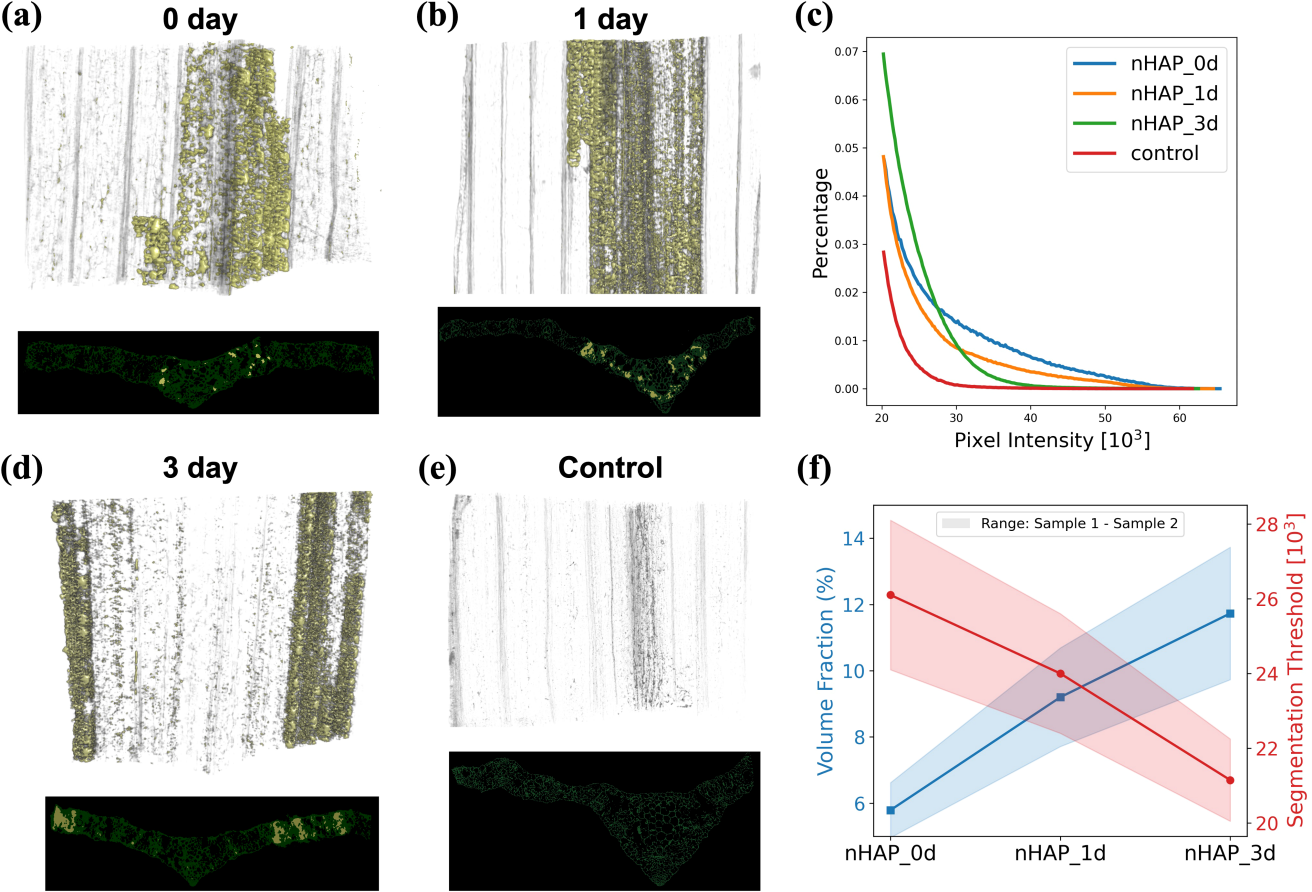
Micro-CT volume renderings and one corresponding virtual cross section (transverse tomographic slice) of plants infiltrated with nHAP for treatment times from 0 to 3 days **(a, b, d)**. A plant of the control group infiltrated only with the citrate solution **(e)**. The histogram **(c)** represents the voxel intensity values in all four samples associated to regions labeled with NPs. Finally, in **(f)** is depicted the volume fraction of NP-labeled regions within the leaf samples across different experimental groups. For each group of experimental conditions, two samples were scanned. The shaded areas of the curves represent the segmentation thresholds and volume fractions of the two samples, respectively. The Micro-CT volume renderings of the other group of samples can be found in the supplementary materials.

SAXS and XRF mapping results in two-dimensional images of the plant leaf with the signal integrated over the third dimension. This third dimension can be unfolded by deploying Micro-CT which produces three dimensional images of the leaf structure as shown in **Figure 6**. This allows precise localization of NP clusters in 3D and simultaneously visualization of the native plant microstructure. To quantify the regions containing NP clusters, we have employed a semi-automated threshold segmentation method to label in yellow the regions containing the NP clusters and in green the native plant structure. Associated to each volumetric image, a virtual cross section is shown in **Figure 6**. To demonstrate the robustness, we also scanned another set of replicates under the same conditions. The corresponding 3D volumetric images and 2D virtual section images are provided in the supplementary materials. From the results we observe that there is no significant decrease in image intensity of the NP rich regions between day 0 and day 1. The high Z-number cerium attached to the NPs is the significant contributor to the image intensity, therefore the constant image intensity means that this element did not translocate in this time frame. On the contrary, when the lighter elements, like phosphorus transition into ionic forms as NPs dissolved, the image intensity will not be significantly affected. In **Figure 6c**, we plotted the histograms of images at all time steps, only taking into account the voxel values in the regions containing aggregated NP solutions. This will allow a quantitative description of the processes. From the histogram comparisons, we can observe that from day 0 to day 1, and then to day 3, the signal distribution gradually shifts to the left, reflecting the progressive dispersion of NPs. This is an essential piece of information to avoid false or over interpretation the SAXS results. In particular we can this way confirm that the unexpected increase at day 3 in SAXS signal at the outer regions of the sample (**Figure 5**) is connected to the fact that NPs or their dissolved constituents like Ce, are more dispersed as separated NP clusters will generate stronger SAXS scattering signals at similar concentrations.

To further illustrate in a quantitative manner the dispersion process of the clusters, we calculated the volume fraction of the regions labeled as NP clusters within the plant samples. This is defined as the ratio of the number of voxels identified as containing NPs (based on their increased intensity) to the total number of voxels in the reconstructed plant sample volume. The segmentation threshold of NPs reflects their packing density: more densely packed aggregates result in higher thresholds due to stronger attenuation and phase-shift contributions in tomographic imaging. In contrast, more dispersed aggregates are associated with lower thresholds. Consequently, denser packing corresponds to a smaller volume fraction, while greater dispersion leads to a larger one. The results in **Figure 6f** show that the segmentation threshold of NP-induced densified regions gradually decreases over time, while the corresponding volume fraction continues to increase. This result also confirms that the NP clusters become more dispersed over time, transitioning from initially tight clusters to more separated clusters.

### 3.3 Pilot study of live plants

The aforementioned experiments with freeze-dried samples effectively demonstrate the advantages of correlative X-ray imaging in plant studies. However, since the comparison is made between different samples, it inevitably introduces inherent differences between samples and variations in infiltration operations, setting strict boundaries on the depth of the results interpretation. We believe that the path forward is to conduct studies on live plants and by this eliminate the issue of individual variability.

We performed a pilot study on live plants in form of single SAXS imaging experiments on two groups of infiltrated plants. As shown in the previous solution experiments, ZnO@MSN and MnO NPs exhibit lower scattering signals compared to nHAP spiked with Ce. However, these are highly relevant NP systems for the development of nano-fertilizers (Gao et al., 2021; Pinna, 2024; Husted et al., 2023). So we chose these NPs to validate the possibility to apply our imaging approach to this challenging system. As described in Section 2.2, SAXS data recorded on live plants were processed as extracting total scattering and scattering anisotropy maps.

**Figure 7:**
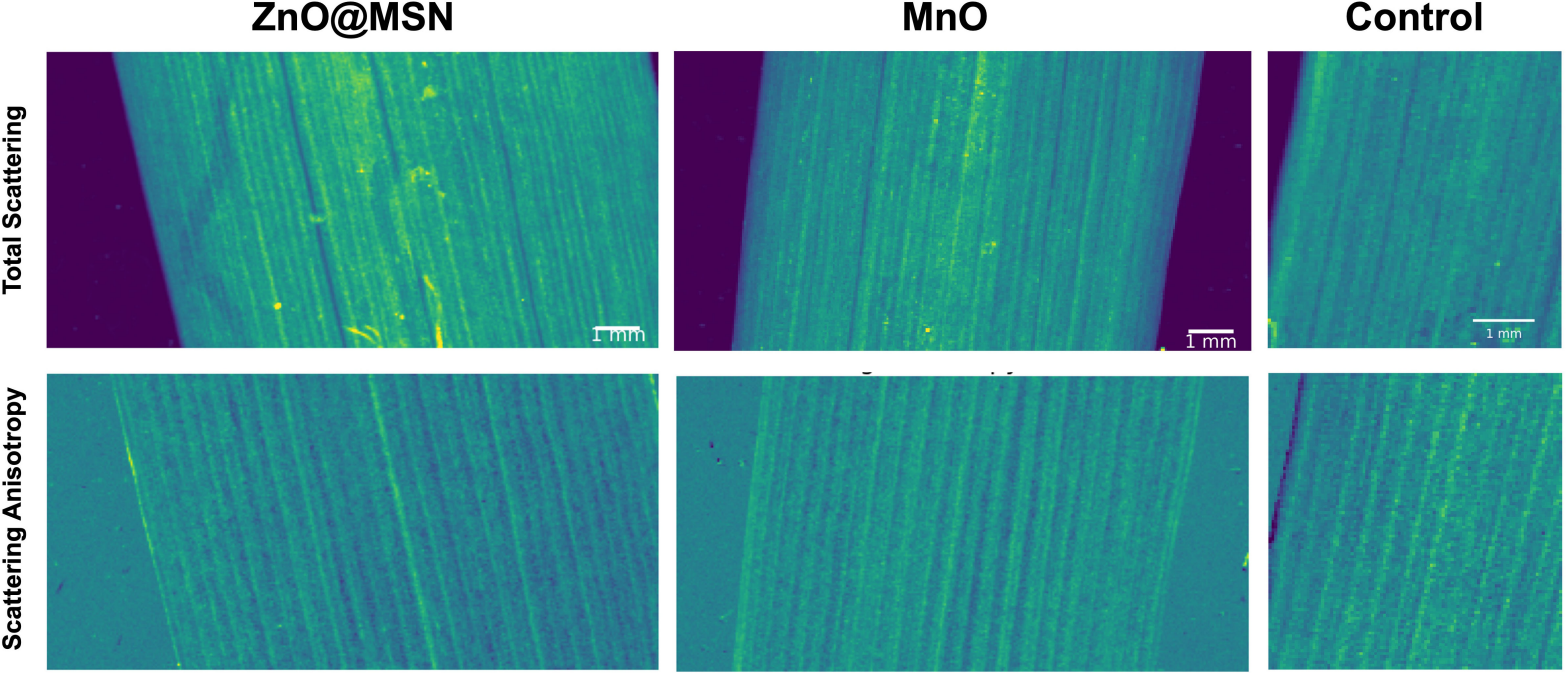
The total scattering intensity and scattering anisotropy maps of the live plants infiltrated with ZnO@MSN and MnO nanoparticle solutions, and one of the control group just infiltrated with the citrate solution.

The total scattering maps of live plant leaves are shown on **Figure 7**. We observed that compared to the control group, for MnO and mainly for ZnO@MSN multiple spots with high scattering signal are visible. After excluding the cellulose structure regions identified in the scattering anisotropy images, the remaining bright spots can be attributed to NP clusters. Additionally, a comparative analysis reveals that samples infiltrated with ZnO@MSN NPs exhibit a stronger scattering signal than those treated with MnO NPs, which is also consistent with our validation study using solutions of various concentrations in **Figure 3**.

Although the SAXS signal from NPs in the live plant samples appear significantly weaker compared to those in the freeze-dried samples, the contrast can be further enhanced by utilizing NPs with stronger scattering signal, increasing particle concentration or incorporating heavy metal elements.

## 4 Discussion

**Figure 8:**
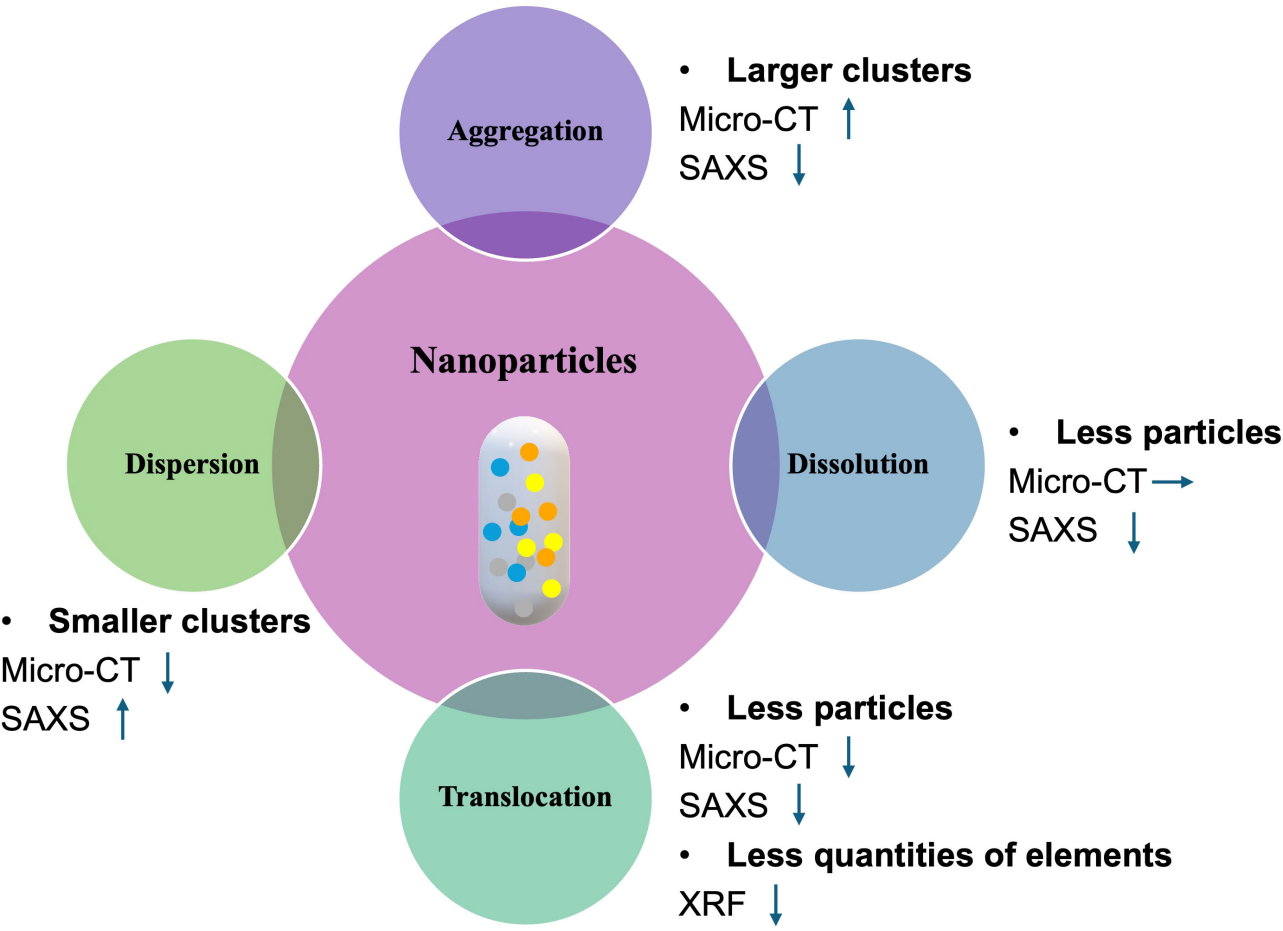
Diagram of the relationship between nanoparticle processes and X-ray imaging signals. Possible subsequent reactions after nanoparticle entry are depicted, including aggregation, dispersion, dissolution, and translocation. Each process leads to distinct intensity variations in different X-ray imaging signals, with upward, downward and rightward arrows indicating signal enhancement, attenuation and stability, respectively.

Given that NPs may undergo various transformations after entering plant tissues, including aggregation, dispersion, dissolution, and translocation (as illustrated in **Figure 8**), we employed a multimodal imaging approach integrating Micro-CT for 3D structural analysis and XRF for elemental mapping alongside SAXS for detecting nanoscale structural signals to differentiate between these processes. In the case of NP aggregation, the formation of larger particle clusters increases X-ray absorption, thereby enhancing the Micro-CT signal. Simultaneously, the growth in particle size reduces the scattering angle range and intensity in SAXS, resulting in a diminished scattering signal. In contrast, dissolution involves the breakdown of NPs into their ionic constituents. This transformation has minimal influence on X-ray attenuation, and thus the Micro-CT signal remains largely unchanged. However, the loss of particulate structure leads to a measurable decline in the SAXS signal. Translocation, defined as the movement of NPs away from the initial site of infiltration, results in decreased X-ray absorption and scattering intensity, producing signal reductions in both Micro-CT and SAXS. This spatial redistribution is also captured by XRF, which detects a corresponding decline in local elemental concentration. Conversely, dispersion, the fragmentation of NP clusters into smaller, more evenly distributed particles, reduces overall X-ray attenuation, weakening the Micro-CT signal. However, once dispersed, the NPs become separated and have a larger interfacial area with the surrounding medium, which enables them to generate stronger SAXS signals. By analyzing these complementary signal patterns across SAXS, Micro-CT, and XRF, we are able to more accurately discern the specific processes influencing NP behavior within plant tissues. This integrative approach enables a more comprehensive and mechanistic understanding of NP fate following foliar infiltration.

By cross-comparing freeze-dried samples analyzed using the three complementary X-ray imaging techniques, we progressively reconstructed the sequence of transformations that NPs undergo following foliar infiltration. Immediately after infiltration, NPs form dense, high-concentration clusters at the injection site. Over the subsequent days, nHAP particles infiltrated into the leaf tissue gradually dissolve and disperse into surrounding regions. This dissolution process is evidenced by the progressive attenuation of the SAXS scattering signal, indicating a loss of particulate structure over time. As the NPs dissolve, their constituent elements, including phosphorus and cerium, are released. Phosphorus, being a highly mobile element in plants, redistributes to distal tissues, whereas cerium remains largely localized to the treated area. This behavior is corroborated by XRF data, which show a declining phosphorus signal intensity over time alongside a relatively stable cerium signal. These observations align with established principles in plant physiology concerning ion mobility: elements such as phosphorus and nitrogen are readily translocated within the plant to support growth in younger sink tissues, whereas others, including calcium and manganese, are comparatively immobile and tend to remain in the tissue where they were initially deposited. Similar immobility is observed for non-essential elements such as cerium and europium, which exhibit limited capacity for long-distance transport within the plant system. These findings not only validate the imaging approach used in this study but also provide valuable insights into the differential mobility and fate of both essential and non-essential elements following NP infiltration.

The results presented above underscore the strong potential of correlative X-ray imaging as a non-destructive method for visualizing NPs within plant tissues. This multimodal approach enables comprehensive analysis of NP behavior by integrating information at the particulate, elemental, and three-dimensional morphology levels. While each technique has individual limitations, their combined application allows for a more holistic understanding that cannot be achieved through any single modality alone. CT relies on the electron density of the sample, requiring a trade-off between radiation dose and resolution. While Micro-CT provides valuable three-dimensional structural information, it remains fundamentally limited to morphological characterization and cannot yield specific chemical composition or elemental distribution data. To address this constraint, XRF was incorporated, enabling two-dimensional mapping of multiple elemental distributions across large sample areas. However, XRF inherently lacks the capability to directly characterize nanoscale particulate features - a critical gap that is specifically addressed in this work through the newly introduced SAXS imaging method. Similar to Micro-CT and XRF, SAXS is also capable of imaging large-scale specimens rather than being limited to surface analysis or localized micro-sections. In our experiments, the primary limitation of SAXS is the strong scattering background arising from the intrinsic structural plant structure. It can be challenging to isolate the signal attributed to the infiltrated NPs. This is of specific concern for live plant imaging where in addition the radiation dose limits the length of the exposure. Based on our pilot study we expect that future live imaging studies have a potential to succeed if the acquisition protocol will follow the concept of first acquiring background SAXS images of plant specimens prior to NP infiltration. Through direct subtraction of the pre-infiltration plant structural signals, the NP-specific SAXS signal contribution can be effectively isolated and quantified. This differential approach would enable more accurate characterization of NP distributions within complex plant matrices. A longitudinal study of this type will require a well-designed sample environment and careful pre-study of radiation damage for the specific plant system. To this end, we investigated how the SAXS signal changes as a function of exposure dose. We observed notable structural changes beginning at around 0.5 seconds of exposure, which led us to limit the exposure time to 50 ms in our experiments. Based on this setting, the dose per exposure is calculated as:

(1) $$D=\frac{I_{0}Et}{A}\frac{\mu }{\rho }\approx 36\text{ kGy},$$

where *I*_0_ ≈ 10^13^ photons/s is the incident X-ray flux at ForMAX (Nygård et al., 2024), the exposure time *t* = 50 ms, the focal spot area *A* = 50 × 50 *µ*m^2^ and the X-ray mass attenuation coefficient *µ/ρ* ≈ 0.56 cm^2^/g at the energy *E* = 20.129 keV. This calculated dose in Equation 1, is close to the upper limit reported by (Jones et al., 2020) for fresh leaves, and well above the threshold for the onset of detectable radiation damage, which is around 4 kGy. Therefore, longitudinal SAXS imaging of the a live sample requires further optimization. One opportunity presents itself by simply using X-rays with shorter wavelength, corresponding to X-ray energies above 30 keV. The benefit of this approach was recently demonstrated for nano-imaging of living plant tissues (Kristensen et al., 2025). This will reduce X-ray absorption and potentially allow multiple scans (Spiecker et al., 2023). However, accessing the same *q*-range at higher energies would require a longer beamline than currently available at ForMAX. As an alternative, we plan to perform lower-dose scans using a laboratory-based SAXS system (Xeuss 3.0, Xenocs S.A) at DTU.

Future developments will equally include an expanded imaged volume of the plant, taking full advantage of the fact that the methods we deployed are inherently not restricted by field of view. Such an improvement will likely lead to the visualization of the spatial and temporal distribution of both undissolved NPs and dissolved ionic species along major vascular pathways and into surrounding tissues—including leaves, stems, and roots. By applying the correlative imaging framework developed here, we aim to reconstruct NP transport dynamics in both space and time, providing a powerful tool for understanding nano-fertilizer behavior *in planta*. Beyond nano-fertilizers, this proposed multimodal X-ray imaging strategy holds broader potential for investigating other NP-based plant delivery systems, such as those used for pesticides, genetic materials, or bioactive compounds.

An additional area for methodological improvement involves the development of more stable and reliable reference markers to support accurate co-registration of multi-modal images. In the present study, the lack of robust spatial markers presented challenges for image alignment. To improve spatial correlation in future experiments, we plan to introduce durable registration strategies, including the use of intrinsic sample features (e.g., leaf edges, corners, midrib tips) or artificial fiducials such as micro-perforations. These techniques will facilitate consistent alignment across different imaging modalities and time points. For freeze-dried samples in particular, methods inspired by herbarium specimen preservation, such as heat-sealing dried leaves in plastic laminate, could enhance sample stability and handling during imaging. Moreover, in future live imaging, potential motion artifacts caused by the plant’s own physiological activities also need to be considered.

In this study, we conducted a series of dilution experiments on three types of pure NP solutions. The next logical step would be systematically investigate the detection limits for various NP types using this multimodal X-ray imaging approach in both freeze-dried and live plant tissues. This will allow imaging based comparisons across a concentration gradient and help establish a quantitative relationship between signal intensity and local NP concentration. Ultimately, this will enable semi-quantitative or quantitative mapping of NP distribution and dynamic changes within plant tissues over time.

## 5 Conclusion

Monitoring the distribution of NPs inside plants is essential for elucidating their interactions with plant systems and for the rational design of nano-carriers aimed at targeted nutrient delivery. In this study, we evaluated the limitations of existing imaging techniques and proposed a correlative imaging strategy employing three complementary X-ray modalities to visualize plant tissues infiltrated with NP formulations. Notably, this work introduces SAXS imaging to the field for the first time. Unlike conventional element tracing techniques, which are generally restricted to surface or thin-section analysis, SAXS leverages the high penetration depth of X-rays to probe nanoscale structures within large sample volumes. By integrating SAXS with Micro-CT, we achieved enhanced three-dimensional localization of NP clusters. This structural information was further complemented by XRF imaging, which provided spatially resolved elemental distributions, thereby enabling the reconstruction of nutrient translocation pathways. Together, these techniques offered a multidimensional understanding of NP behavior and transformation within plant tissues.

In this pilot study, we demonstrated the dissolution behavior of NPs using freeze-dried plant specimens. Preliminary SAXS imaging on live plants further confirmed that NPs can generate detectable signals above background levels. While current freeze-dried plants were infiltrated with high-concentration NP solutions containing high atomic number (Z) elements, SAXS imaging is not inherently limited to such materials. Rather, its broader application hinges on the ability to accurately subtract background scattering signals from plant tissues. Future live plant experiments will incorporate pre-infiltration baseline imaging to enable background subtraction, allowing for broader application to low-Z and low-concentration NP systems.

In summary, the proposed correlative X-ray imaging strategy provides a powerful and non-destructive tool for studying NP dynamics within complex plant matrices. Beyond its immediate utility for imaging, this methodological advancement offers valuable insights for mechanistic studies in emerging domains such as nano-fertilizer development and precision agriculture.

## Data Availability

The datasets presented in this study can be found in online repositories. The names of the repository/repositories and accession number(s) can be found below: https://figshare.com/s/33ee8388a36600fe98d5.
