# Correction: Correlative X-ray Imaging to Reveal the Dissolution of Nanoparticles and Nutrient Transport in Plant Foliar Fertilization

**DOI:** 10.3389/fpls.2026.1842617

**Published:** 2026-04-20

**Authors:** Chengpeng Wu, Emil V. Kristensen, Francesco Minutello, Augusta Szameitat, Søren Husted, Rajmund Mokso

**Affiliations:** 1 Department of Physics, Technical University of Denmark, Lyngby, Denmark; 2 Plant and Environmental Sciences, University of Copenhagen, Frederiksberg, Denmark

**Keywords:** SAXS, XRF, Micro-CT, plant imaging, foliar fertilization, dissolution of nanoparticles, nutrient transport

In the published article “Szameitat, A.E., Sharma, A., Minutello, F., Pinna, A., Er-Rafik, M., Hansen, T.H., Persson, D.P., Andersen, B. and Husted, S., 2021. Unravelling the interactions between nano-hydroxyapatite and the roots of phosphorus deficient barley plants. Environmental Science: Nano, 8(2), pp.444-459. doi: 10.1039/d0en00974a” was not cited in the article. The citation has now been inserted in Section 2 **Materials and methods**, *2.1 Nanoparticles and plant samples*, Paragraph 1 and should read:

“This study primarily involves three types of NPs, including hydroxyapatite (nHAP), mesoporous silica with ZnO (ZnO@MSN), and manganese oxide (MnO), selected for their relevance in the field of nanofertilization (Hadri etal.,2024). The chemical compositions and structural characteristics of the NPs used in this study are summarized in **Table 1**. Particle synthesis and characterization are described in Burve et al. (2024); Gao et al. (2021); Pinna (2024); Szameitat et al. (2021). To enhance the X-ray imaging contrast of nHAP particles, we employed a spiking strategy by incorporating two heavy metal elements, cerium (Ce) and europium (Eu), into their structure.”

**Table 1:** Chemical compositions and structural parameters of the NPs used in this study.

Name	Components	Geometric parameters	Description
nHAP-1	Ca10(PO4)6(OH)2	Length: 28 nm; Width: 16 nm	Crystalline nano-hydroxyapatite
nHAP-2	Ca10(PO4)6(OH)2+ Ce + Eu	Length: 40 ± 12 nm; Width: 13 ± 5 nm	Crystalline nano-hydroxyapatite doped with cerium and europium
ZnO@MSN	SiO2 + ZnO	Core radius: 33 nm; Shell thickness: 12 nm	ZnO encapsulated in mesoporous SiO2
MnO	(C3H4O2)n + MnO	Radius: 12 nm	poly(acrylic acid)-coated Mn oxide

In the table, nHAP-1 is used in the solution experiments in Section 3.1, while nHAP-2 is used in the freeze-dried leaf experiments in Section 3.2.

In the published article, there was an error in **Table 1** as published. With the collaborator’s reminder, the author realized that the two types of nHAP nanoparticles used in the experiments were synthesized by different methods and had variations in their geometric parameters. Therefore, the relevant parameters of the two particles were updated in **Table 1**. The corrected **Table 1** and its caption “Chemical compositions and structural parameters of the NPs used in this study.” appear below.

In the published article, there was an error. To express our gratitude for the collaborator’s reminder, we have added the following content to the **Acknowledgements** section.

A correction has been made to **Acknowledgements**, Paragraph 1. This sentence previously stated:

“The authors would like to thank the scientists Dr. Kim Nygård and Dr. Mira Viljanen at the ForMAX beamline at MAX IV for their valuable help with the synchrotron SAXS measurements. And thank Dr. Grmay Hailu Lilay and Mr. Tommaso Angeletti for providing the BY-2 cell samples. We acknowledge support from the Danish National Facility for Imaging with X-rays, DANFIX.”

The corrected sentence appears below:

“The authors would like to thank the scientists Dr. Kim Nygård and Dr. Mira Viljanen at the ForMAX beamline at MAX IV for their valuable help with the synchrotron SAXS measurements. We thank Dr. Grmay Hailu Lilay and Mr. Tommaso Angeletti for providing the BY-2 cell samples, and Dr. Regina Burve and Dr. Jean-Claude Grivel for providing the nHAP nanoparticles. We acknowledge support from the Danish National Facility for Imaging with X-rays, DANFIX.”

The original article has been updated.

