# Supplementary material for: Correlative X-ray imaging to reveal the dissolution of nanoparticles and nutrient transport in plant foliar fertilization
Source: Front Plant Sci. 2025 Jun 24;16:1610402. doi: 10.3389/fpls.2025.1610402 (PMC12235465; doi:10.3389/fpls.2025.1610402)
Supplement: Supplementary file 1 [file DataSheet1.pdf]

Supplementary Material

1 SUPPLEMENTARY FIGURES

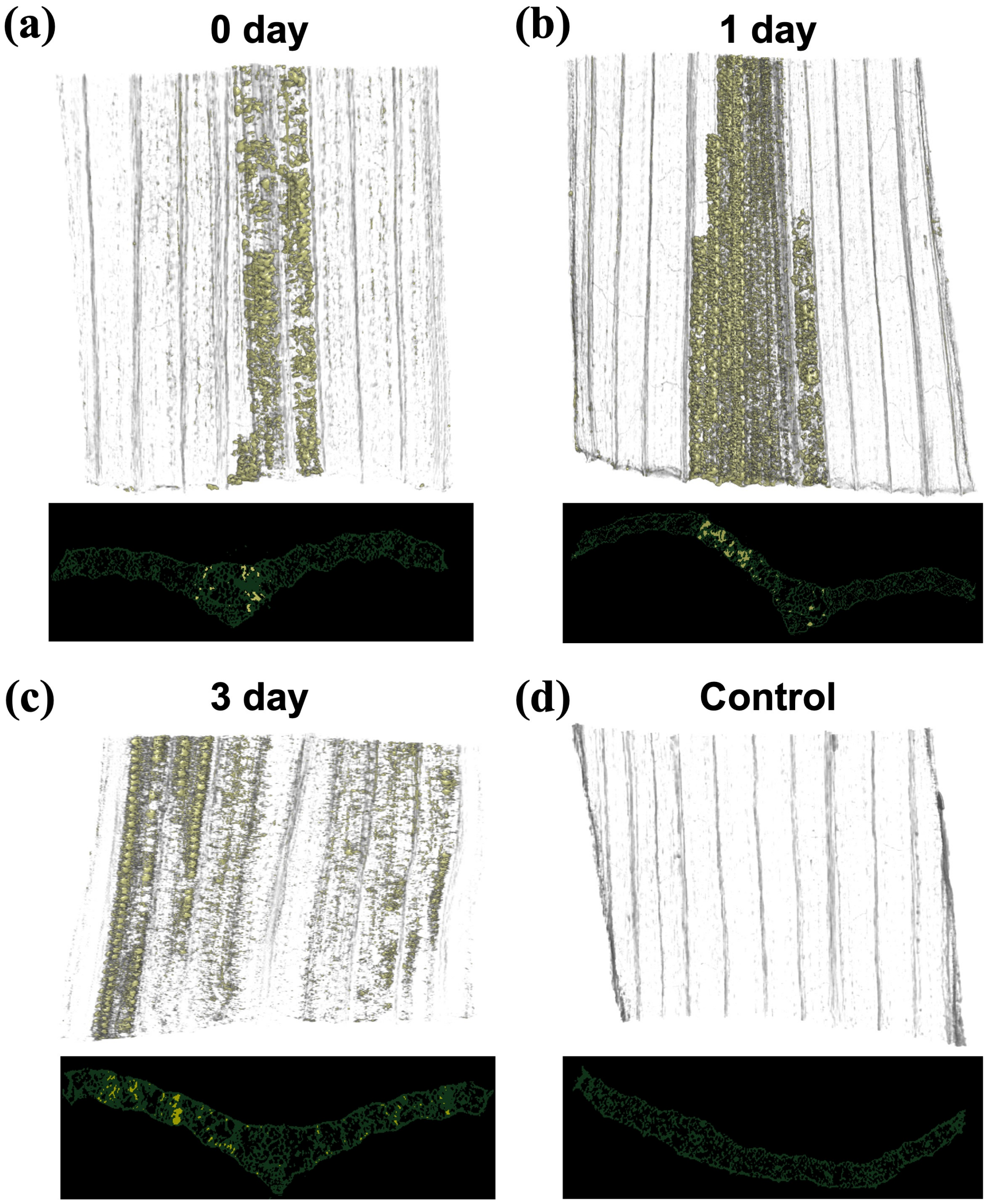

Figure S1. Micro-CT volume renderings and one corresponding virtual cross section (transverse tomographic slice) of the other group of freeze-dried plants infiltrated with nHAP for treatment times from 0 to 3 days (a,b,c). A plant of the control group infiltrated only with the citrate solution (d).
